# Age-related compaction of lens fibers affects the structure and optical properties of rabbit lenses

**DOI:** 10.1186/1471-2415-7-19

**Published:** 2007-12-20

**Authors:** Samer Al-khudari, Sean T Donohue, Walid M Al-Ghoul, Kristin J Al-Ghoul

**Affiliations:** 1 Department of Anatomy and Cell Biology, Rush University Medical Center, Chicago, IL, USA; 2 Department of Biological Sciences, Chicago State University, Chicago, IL, USA; 3 Department of Ophthalmology, Rush University Medical Center, Chicago, IL, USA

## Abstract

**Background:**

The goal of this investigation was to correlate particular age-related structural changes (compaction) to the amount of scatter in rabbit lenses and to determine if significant fiber compaction occurred in the nuclear and inner cortical regions.

**Methods:**

New Zealand White rabbits at 16–20 months old (adult; n = 10) and at 3.5–4 years old (aged; n = 10) were utilized for this study. Immediately after euthanising, scatter was assessed in fresh lenses by low power helium-neon laser scan analysis. Scatter data was analyzed both for whole lenses and regionally, to facilitate correlation with morphometric data. After functional analysis, lenses were fixed and processed for scanning electron microcopy (SEM; right eyes) and light microscopy (LM; left eyes). Morphometric analysis of SEM images was utilized to evaluate compaction of nuclear fibers. Similarly, measurements from LM images were used to assess compaction of inner cortical fibers.

**Results:**

Scatter was significantly greater in aged lenses as compared to adult lenses in all regions analyzed, however the difference in the mean was slightly more pronounced in the inner cortical region. The anterior and posterior elliptical angles at 1 mm (inner fetal nucleus) were significantly decreased in aged vs. adult lenses (anterior, p = 0.040; posterior, p = 0.036). However, the average elliptical angles at 2.5 mm (outer fetal nucleus) were not significantly different in adult and aged lenses since all lenses examined had comparable angles to inner fetal fibers of aged lenses, i.e. they were all compacted. In cortical fibers, measures of average cross-sectional fiber area were significantly different at diameters of both 6 and 7 mm as a function of age (p = 0.011 and p = 0.005, respectively). Accordingly, the estimated fiber volume was significantly decreased in aged as compared to adult lenses at both 6 mm diameter (p = 0.016) and 7 mm diameter (p = 0.010).

**Conclusion:**

Morphometric data indicates that inner cortical fibers undergo a greater degree of age-related compaction than nuclear fibers. Increased scatter appears to be only tentatively correlated with regions of fiber compaction, suggesting that it is simply one of an array of factors that contribute to the overall decreased transparency in aged rabbit lenses.

## Background

It is generally accepted that the formation of age-related cataracts is a multifactorial process that results from an acceleration and/or accentuation of normal aging changes within the lens. Indeed, structural, biochemical and physiological studies of age-related cataract have all uncovered a multitude of senescent changes affecting lens fibers at both the molecular and cellular levels. Molecular alterations include, but are not limited to, posttranslational protein modifications resulting from glycation, phosphorylation, deamidation, and especially oxidation [1-3]. These modifications affect protein conformation, and may also initiate cross-linking and aggregation[4] and hence are likely to negatively impact function.

At the cellular level, numerous structural modifications have been documented that are likely to contribute to light scattering. Fiber folds and breaks[5], multi lamellar bodies[6,7], extracellular space deposits [8,9], malformed or excessive suture sub-branches[10], syneresis and micro-phase separation of fiber cytoplasm [11-14] and fiber compaction [15-17] have all been observed in aged human lenses and proposed as potential sources of light scatter contributing to age-related cataract formation. Compaction of lens fibers is of particular interest because it has been shown to begin before middle age[18], thus potentially contributing to presbyopia as well as age-related cataracts.

Our prior studies have shown that aging human lenses exhibit specific structural changes that contribute to significant, measurable lens compaction, especially in nuclear regions[16,17]. This compaction was markedly greater in both age-related nuclear cataracts and late-onset diabetic cataracts as compared to aged normal (non-cataractous) lenses. In a subsequent study of rabbit lenses, it was established that significantly more scatter occurs as a function of age[19]. Additionally, structural parameters indicated that age-related compaction may have occurred in rabbit lenses. Taken together, these studies lead us to hypothesize that compaction of lens fibers occurs along the visual axis and may be a factor contributing to increased light scatter as a function of age.

Objective measures of lens function can be obtained using the Scantox *In Vitro *Assay System which provides assessments of both focal variability and transmittance in freshly dissected, unfixed lenses. Due to the requirement for unfixed tissues, direct assessment of human lenses is problematic, requiring the use of an animal model. The rabbit lens is suitable for several reasons. First, both human and rabbit lenses possess branched suture patterns. However, rabbit lenses feature a "line" suture pattern, whereas human lenses feature a "star" suture pattern (see [20,21] for reviews on lens suture formation.). Thus, rabbit lens fiber arrangement can be considered a simplified version of the more complex fiber organization in human lenses. Second, rabbit lenses are closer in size and sphericity to human lenses than other commonly used rodent lenses such as mouse and rat. Lastly, our prior study of functional lens parameters [19] utilized rabbit lenses, and therefore provides a baseline for comparison in the present investigation.

The first systematic study of normal rabbit lens ultrastructure was conducted by Harding *et al*. [22] and provided both quantitative and qualitative data on lens fibers at different locations within the young adult rabbit lens. A subsequent investigation [23] reassessed rabbit lens fiber ultrastructure and membrane specializations in infant and young adult rabbits, thus yielding information on structural alterations that occur during development and growth. Numerous other investigators have studied various aspects of rabbit lens ultrastructure including fiber organization [24-26], fiber processes [27,28], sutural anatomy[29], fiber development[30] and accommodative range[31], thus providing a comprehensive picture of lens structure in this important animal model. In the present study, we have utilized some of the same techniques as earlier investigators (scanning electron microscopy, light microscopy, biometry and laser scan analysis) to compare adult and aged rabbit lenses in order to provide data on senescent changes in lens fibers. Specifically, our initial aim was to correlate particular structural changes which occur with aging (i.e. compaction of central lens fibers along the visual axis) to the amount of light scatter in rabbit lenses. We demonstrated a limited amount of compaction in nuclear regions as well as indications that compaction was more pronounced in the periphery of the lens nucleus, adjacent to the cortex. Consequently, our second aim was to examine the inner cortical fibers for evidence of age-related compaction. Our results revealed that a significant reduction of cortical fiber volume occurs in rabbit lenses as a function of age.

## Methods

### Lenses

All animals were handled in accordance with the ARVO Statement for the Use of Animals in Ophthalmic and Vision Research and in compliance with Institutional Animal Care and Use Guidelines. We utilized a total of 20 New Zealand white rabbits in this investigation. Rabbits raised under laboratory conditions reach sexual maturity between 4 and 6 months of age and have an average life span of 5 to 8 years[32]. Accordingly, for this study, rabbits16–20 months old (n = 10) were designated 'adult' and rabbits 3.5–4.5 years old (n = 10) were designated 'aged'. Animals were euthanized by intracardial injection of sodium pentobarbital (398 mg/ml at 1.0 ml/10 lb body weight). Immediately after euthanising, eyes were enucleated from the orbit and lenses were dissected from the eyeball in M199 culture media (supplemented with Earle's salts and 8% fetal bovine serum) at 37°C. Fresh unfixed lenses from the right (OD) eyes were immediately assessed by low power helium-neon laser scan analysis as detailed below. These lenses were then fixed, and prepared for scanning electron microcopy (SEM). Left eye (OS) lenses were enucleated, fixed and processed for light microscopy (LM).

### Laser Scan Analysis

**Figure 1:**
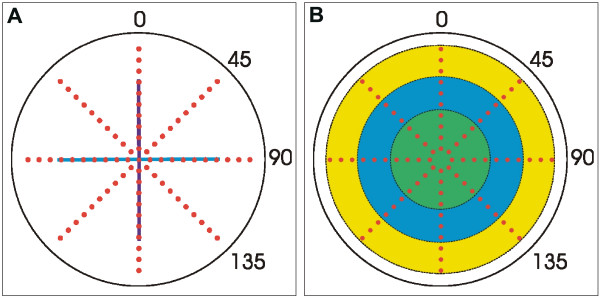
Diagrams showing how the functional assessment of fresh lenses and analysis of the results was performed. A. The location of laser penetrations during laser scan analysis of fresh rabbit lenses is represented by filled red circles. Four scan series' were done for each lens at 0°, 45°, 90° and 135° to provide scan data along the anterior (dark blue, vertical line) and   posterior (light blue, horizontal line) lens sutures, as well as maximally    off of the sutures.   B. For analysis the scan data was divided into three groups: outer scans (yellow ring), intermediate scans (blue ring) and inner scans (green ring) which corresponded to specific ranges of equatorial diameter. Specifically, the outer region contained measures from 10 mm to 7.6 mm, the intermediate region contained measures from 7.5 mm to 5.5 mm and the inner region contained measures that were < 5.4 mm.

Within five minutes of animal euthanasia, the mean back vertex distance (average BVD) and scatter were assessed for OD lenses using the Scantox *In Vitro *Assay System (Harvard Apparatus Inc.) as described previously[19]. Briefly, lenses were placed in specially-designed chambers of glass and silicon rubber containing fresh M199 culture media at 37°C (supplemented as detailed above) for scanning. Both anterior and posterior surfaces of the lens were bathed in culture media at all times. Lenses were suspended within the glass chamber on a size-appropriate, beveled washer which supported the equatorial rim, and oriented so that lasers pass through the lens from the anterior to the posterior. (See [19] for a schematic diagram of the laser scan apparatus). All lenses were scanned with 4 series of laser beams as shown in Figure 1a, so that the first scan series (at 0°) passed along the posterior suture. Three additional series of scans were done at 45°C, 90°C and 135°C, to provide scan data along the anterior suture (90°C) and maximally off of the sutures (45°C and 135°C). Each series of laser scans consisted of twenty penetrations at equal increments passed along the lens, resulting in a total of eighty objective measurements per lens. Measurements of transmittance were made by two digital cameras that automatically record the actual position, slope and intensity of the laser beams passed through the lens. Transmittance was measured as the total pixel value of the refracted laser beam through a given point in a lens. The main source of differences in transmittance is probably due to scatter, although absorbance also contributes to transmittance values.

Statistical calculations (Student's t-test) were performed to determine if scatter in aged rabbit lenses was significantly different from adult rabbit lenses. Both overall and regional scatter was evaluated (using Microsoft Office Excel 2003; Microsoft Corp., Redmond, WA, U.S.A.). Overall scatter comprised all of the measures from the four scan series across the entire lens diameter. Regional measures were derived by dividing each scan series into three groups: outer scans, intermediate scans and inner scans (Fig. 1b). Because the Scantox *In Vitro *Assay System provides the precise offset distance (from the lens center) of each laser penetration (as well as the back vertex distance and scatter measures), it is possible to correlate each individual measure with an anatomical location within the lens. We utilized this data to separate laser scan measures into the three sets listed above wherein each set corresponded to a specific range of equatorial lens diameters. Specifically, the outer region contained measures from 10 mm to 7.6 mm, the intermediate region contained measures from 7.5 mm to 5.5 mm and the inner region contained measures that were < 5.4 mm (Fig. 1b and Table 1). These divisions were chosen in order to correlate the scatter data with morphometric data gathered from structural analyses.

**Table 1:** Average Scatter in Whole Fresh Lenses

	**Average Scatter ± SEM**		
		
**Region**	**Adult Lenses**	**Aged Lenses**	**p Value**
Total Scans (Whole Lenses)	182.94 ± 10.99	253.72 ± 18.94	0.012*
Outer Scans (10–7.6 mm)	155.70 ± 2.66	224.35 ± 13.07	0.014*
Intermediate Scans (7.5–5.5 mm)	181.25 ± 7.95	253.70 ± 18.83	0.009*
Inner Scans (< 5.4 mm)	193.78 ± 15.00	264.01 ± 20.20	0.024*

* Statistically significant

### Lens fixation

All lenses were fixed for 24 hr at room temperature in 10% neutral buffered formalin (in 0.1 M phosphate buffer), then washed in buffer and further fixed for 3–5 days in 2.5% glutaraldehyde (in 0.12 M sodium cacodylate buffer, ph7.2) at room temperature with fresh fixative changes daily. These fixation conditions have been empirically tested and shown to result in negligible osmotic stress in rabbit lenses [33-36]. After overnight washing in 0.2 M sodium cacodylate buffer, the equatorial and axial anterior-posterior (A-P) dimensions were measured under a dissecting microscope. Lenses were then processed for either SEM or LM.

### Specimen dissection and processing for SEM

**Figure 2:**
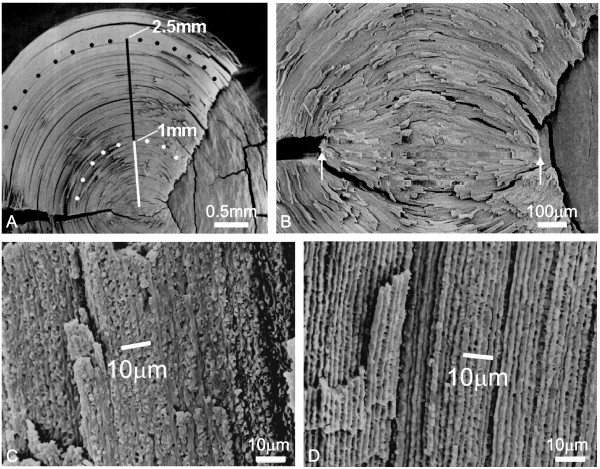
Scanning electron micrographs showing how nuclear morphometry was performed. A. Measurements of elliptical angles in the fetal nucleus (FN) were made on micrographs of split lens nuclei, allowing for exposure of the radial cell columns. A distance of 1 mm was measured from the lens center (white line) and the fetal fibers were traced (white dots). Similarly, a distance 2.5 mm was measured from the lens center (white line + black line) and the fetal fibers at that point were traced (black dots). Angle measurements were made using ellipse templates. B. Higher magnification view of the embryonic nucleus (EN). The anterior-posterior (A-P) axis was measured between the two arrows, which are placed at the anterior and posterior poles of the EN. Note that the borders of the EN are demarcated by the initiation of fetal suture planes. C-D. Micrographs illustrating how the average number of fibers per 10 μm was measured. Fibers at 1 mm from the lens center (along the equatorial axis) were followed to their anterior (panel C) or posterior (panel D) ends and photographed. Multiple measures of the number of fiber ends per 10 μm were made across each micrograph, and then averaged.

Lenses were dissected as previously described[16]. Briefly, successive layers of lens fibers were peeled around the lens circumference until an equatorial diameter of approximately 5 mm was reached. The remaining lens mass was designated as the nucleus, and was split along the anterior suture plane, exposing the radial cell columns from the periphery to the center (Fig. 2A), then processed for SEM. The splitting of lens nuclei resulted in two complementary specimens, thus providing an avenue for additional measurements in cases of specimen damage or artifacts of preparation.

The dissected lens pieces were post-fixed in 1% aqueous osmium tetroxide at 4°C overnight, washed in 0.2 M sodium cacodylate buffer, then dehydrated through a graded ethanol series. After overnight dehydration in 100% ethanol, the ethanol was replaced through a graded ethanol/Freon 113 series to pure Freon 113. Specimens were critical point dried in Freon 23 (Dupont, Wilmington, DE, U.S.A.) in a Balzers CPD 020 (Balzers, Hudson, NH, U.S.A.), secured on aluminum stubs with silver plaster, sputter coated with gold and examined in a JEOL JSM 35 c scanning electron microscope (JEOL USA, Peabody, MA, U.S.A.) at 15 kV. Electron micrographic magnification series' were taken (from 20X to 3000X) for each specimen.

### Nuclear Morphometry

Five structural parameters were defined and measured directly from the SEM micrographs. These were: 1) The A-P axis of the embryonic nucleus (EN) in μm, 2) the ellipsoid angles described by anterior portions of fibers in the fetal nucleus (FN) at 1 mm and 2.5 mm from the lens center, 3) the ellipsoid angle described by posterior portions of FN fibers at 1 mm and 2.5 mm from the lens center, 4) the average number of FN fiber ends in an anterior radial cell column per 10 μm length, 5) average the number of FN fiber ends in a posterior radial cell column per 10 μm length. The presence or absence of accordion-like compaction folds was noted but not quantified.

In keeping with prior structural analyses of crystalline lenses[37,38], the embryonic nucleus was defined as the primary lens fibers, whereas the fetal nucleus was defined as all of the secondary fibers added until birth. All measurements were made directly from scanning electron micrographs and were carried out in a similar manner to previous structural investigations of fiber compaction[16,17]. Specifically, the A-P axis of the embryonic nucleus was measured along the optic axis from the tips of EN fibers adjacent to the initiation of the anterior fetal suture plane to the tips of EN fibers adjacent to the initiation of the posterior fetal suture plane (Fig. 2B, white arrows). In a lens split along the anterior suture, the anterior suture plane appears roughly triangular whereas the posterior suture is visible as a line oriented at a 90°C angle to the anterior plane. Thus the fibers encompassed between these two planes are the primary fibers since, by definition, primary fibers do not form sutures. The ellipsoid angles of fetal nuclear fibers were evaluated at 1 mm and 2.5 mm from the lens center. This was done by measuring the desired distance (1 or 2.5 mm) from the lens center on an SEM micrograph along the equatorial axis of the split lens (Fig. 2A, white and black lines). This line was utilized as the semi-major axis of the ellipse. Fibers at the desired equatorial distance were traced along their anterior and posterior segments and marked (Fig. 2A, white and black dotted arcs). Engineering ellipse templates (Alvin & Co. Inc., Windsor, CT, U.S.A.) were then superimposed over the arcs until an appropriate match was obtained, resulting in a direct measurement of the anterior and posterior elliptical angles. To measure the average number of fibers per 10 μm in anterior or posterior radial cell columns, a distance of 1 mm was measured from the lens center along the equatorial axis of the split lens (Fig. 2A, white line) and marked. These fibers were followed along the anterior or posterior segments, and higher magnification SEM micrographs were taken adjacent to their ends (Fig. 2, C–D). Multiple (4–6) measurements were made on each micrograph by counting the number of fibers in a 10 μm length. Average values were calculated for each sample.

### Specimen dissection and processing for LM

**Figure 3:**
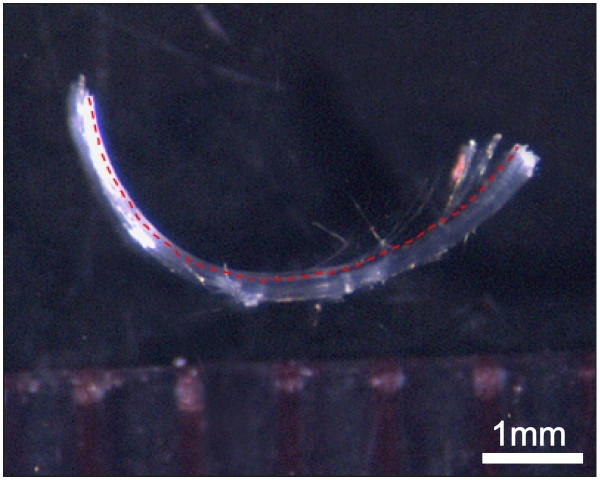
Dissecting microscope picture of a group of straight fibers (i.e. fibers lacking opposite end curvature). Small groups of straight fibers were carefully dissected from the lens at selected locations, specifically at 6 mm and 7 mm equatorial diameter. Measurement of fiber length (dashed line) was made using the digitized photographs and an image analysis program.

After initial fixation, lenses were carefully dissected by removing crescent-shaped groups of fibers at successive depths. Although all fibers are crescent-shaped and extend from the anterior to posterior sides of the lens, only some of these fibers approximate meridians. Specifically, fibers that terminate at the proximal ends of suture branches or at the poles lie entirely within a longitudinal plane and have straight end segments. These are termed 'straight fibers'[39]. In contrast, the remainder of the lens fibers do not lie within a single longitudinal plane; they display opposite end curvature, approximating an 's' shape. At two specific locations, 6 mm and 7 mm equatorial diameter, groups of straight fibers (i.e. those lacking opposite end curvature) were carefully dissected from the lens circumference, retrieved and photographed (Fig. 3). Straight fibers were used to measure fiber length because they are planar (i.e. they do not have opposite end curvature). Because rabbit lenses have line sutures, straight fibers are present at four locations in each growth shell [20]: 0°C, 90°C, 180°C and 270°C. The straight fibers extend either from the proximal end of the anterior suture branches to the posterior pole or from the proximal end of the posterior suture branches to the anterior pole. Thus, we collected 3–4 small groups of straight fibers at both 6 mm and 7 mm equatorial diameter to obtain an average fiber length for each depth. Photographs of straight fibers were scanned, digitized and the length of straight fibers was measured using image analysis software (Scion Image v. beta 4.0.2; Scion Corp., Frederick MD) as shown in Figure 3.

Specimens were sequentially *en bloc *stained with 1% tannic acid and 2% aqueous uranyl acetate (4°C, dark), and then dehydrated through a graded ethanol series to 100% ethanol. Dehydrated specimens were infiltrated with LR White resin and embedded in gelatin capsules, then polymerized at 50°C C for 24 hours. Embedded specimens, visible within the polymerized blocks were examined under a dissecting scope and the equatorial plane was marked on the block to ensure that fibers would be cut in cross-section. Blocks were bisected with a jeweler's saw to obtain fiber cross sections of equatorial segments. Semi-thin (1–2 μm) sections were cut on a Sorvall MT2 ultramicrotome, mounted on glass slides, stained with methylene blue and examined on Nikon Eclipse 80 i microscope (Nikon USA, Melville, NY, U.S.A.) at 60X with 1.6X zoom. Images were recorded using a Q-Imaging Retiga 1300 digital camera in conjunction with Q-Capture v. 2.60 software (both from Quantitative Imaging Corp., Burnaby, BC, Canada), and stored as TIFF images at a minimum of 8-bit quality.

### Cortical Morphometry

Image analysis was performed using Scion Image v. beta 4.0.2 software (Scion Corp., Frederick, MD, U.S.A.). Cross sectional area was measured by delineating the perimeter of individual fiber profiles. Average cross sectional area was determined for each lens using 100–150 measurements. The volume of a fiber can be estimated by using the formula for the volume of a cylinder: volume = cross sectional area X length. This estimate does not take into account the fact that lens fibers taper as they extend anteriorly and posteriorly toward the poles. However, because our preliminary data did not provide any evidence that fiber end thickness was significantly reduced as a function of age[40] and Table 2, the formula for the volume of a cylinder provides a reasonable and consistent method to compare the two age groups in this study. Statistical analysis (Student's t-test) was conducted using Microsoft Office Excel 2003 (Microsoft Corp., Redmond, WA, U.S.A.) to compare both the average cross-sectional area and the estimated volume of adult vs. aged lens fibers at comparable locations of 6 mm and 7 mm equatorial diameter.

**Table 2:** Morphometry of Rabbit Lens Nuclei (Scanning EM)

**Parameter**	**Adult Lenses (n = 9)**	**Aged Lenses (n = 8)**	**p Value**
EN Anterior-Posterior Axis Length (μm)	685.71	670.83	0.710
Anterior FN Elliptical Angles: 1 mm	64.71	56.00	0.040*
Anterior FN Elliptical Angles: 2.5 mm	57.80	57.00	0.816
Posterior FN Elliptical Angles: 1 mm	66.71	57.86	0.037*
Posterior FN Elliptical Angles: 2.5 mm	58.20	58.80	0.837
# Anterior FN ends/10 μm	3.94	4.03	0.804
# Posterior FN ends/10 μm	4.69	4.69	0.995

* Statistically significant

## Results

### Optical Analysis

Laser scan analysis of rabbit lenses was performed within five minutes of euthanasia using the Scantox *In Vitro *Assay system. Complete results of the statistical analysis of laser scan data are presented in Table 1. The average scatter was significantly greater in aged lenses as compared to adult lenses for both overall scatter (p = 0.012) and for each of the regions evaluated separately. It is interesting that the most pronounced difference in the mean scatter was found in the intermediate region (7.5-5.5 mm equatorial diameter) which corresponds to the inner cortical region of the lens.

### Lens Dimensions

Because the rabbit lens, like all vertebrate lenses, grows throughout life[41]measurements were made to assess the age-related changes to overall lens dimensions. In adult rabbit lenses, the average equatorial diameter was 11.35 mm and the average A-P axis length was 7.49 mm. In comparison, aged rabbit lenses had an average equatorial diameter of 12.04 mm and an A-P axis length of 7.92 mm. Thus the equatorial diameter increased by 6.08% and the A-P axis length increased by 5.74%.

### Embryonic and Fetal Nuclear Regions

Following Laser Scan Analysis, lenses were processed for SEM and morphometry was performed using the resultant micrographs. Five structural parameters were defined and measured directly from the SEM micrographs of 9 adult and 8 aged lenses. These were: 1) The A-P axis of the EN in μm, 2) the ellipsoid angles described by anterior portions of fibers in FN at 1 mm and 2.5 mm from the lens center, 3) the ellipsoid angle described by posterior portions of FN fibers at 1 mm and 2.5 mm from the lens center, 4) the average number of FN fiber ends in an anterior radial cell column per 10 mm length, 5) the average number of FN fiber ends in a posterior radial cell column per 10 mm length. Detailed results are presented in Table 2.

The anterior and posterior FN elliptical angles at 1 mm (inner fetal fibers) were significantly decreased in aged vs. adult lenses (anterior, p = 0.040; posterior, p = 0.036). The average elliptical angles of outer fetal fibers (2.5 mm from the center of the EN) were not significantly different in adult and aged lenses since all lenses examined had comparable angles to inner fetal fibers of aged lenses (Table 2), i.e. they were all compacted. This surprising result (less compaction in the oldest inner nuclear fibers as compared to the outer nuclear fibers), prompted additional investigation of fiber structure as a function of age, near the cortical-nucleus interface.

Although the average length of the AP axis in the EN of aged lenses was smaller than in adult lenses, the reduction was not statistically significant. Similarly, when comparing the number of anterior or posterior ends per 10 μm length in aged vs. adult lenses, no significant difference was found.

**Figure 4:**
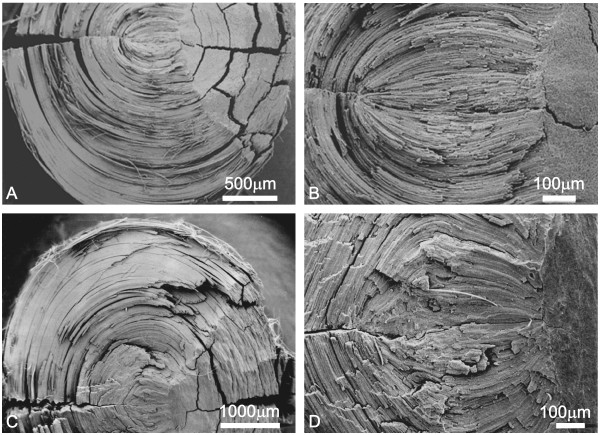
Representative scanning electron micrographs of split rabbit lens nuclei. A-B. Low and medium magnification of adult rabbit lens nuclei. C-D. Low and medium magnification of aged rabbit lens nuclei. It is clear that the EN of aged lenses appeared more disorganized than the EN of adult lenses (compare panels B and D).

**Figure 5:**
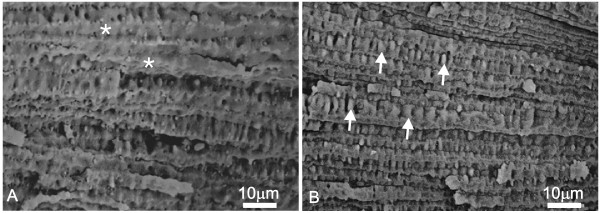
Scanning electron micrographs of surface topology in EN fibers. A. Adult lens. The broad faces of EN fibers are smooth (asterisks) or contain only low-amplitude folds. B. Aged lens. The numerous, accordion-like folds (arrows) occur on virtually every broad face of EN fibers and show variation in frequency and moderate to high amplitude. These folds were present on EN fibers in 5 (of 8) lenses examined and are remarkably similar to accordion-like folds found in the nuclei of aged human lenses.

Representative SEM images of aged and adult rabbit lenses are presented in Figure 4 to illustrate the morphometric results. It is clear that the EN of aged lenses (Fig. 4, C–D) appeared more disorganized than the EN of adult lenses (Fig. 4, A–B). At higher magnification, we examined the surface topology of EN fibers (Fig. 5, A–B). The EN fibers of several aged rabbit lenses displayed high-amplitude, accordion-like folds (Fig. 5B, arrows) analogous to those seen in aged human lenses [16,17]. However, these were not consistently present in all aged rabbit lenses (5 of 8). In comparison, the surface topology of adult EN fibers displayed smooth faces (Fig. 5A, asterisks) or lower amplitude, less frequent accordion-like folds. The accordion-like folds are distinct from other membrane specializations in rabbit lens fibers such as ball and socket interdigitations, flap and imprint protrusions and surface folds and bends (which occur exclusively in the bow region of the intermediate cortex[23]). Low magnification also demonstrates the more compressed appearance of the FN in aged as compared to adult lenses, which resulted in smaller elliptical angles.

### Inner Cortical Region

Because the quantitative data from SEM analysis indicated that compaction occurred in the periphery of the FN adjacent to the cortex, we assessed the inner cortical region for evidence of compaction. SEM analysis comprised measurements as far as 2.5 mm from the lens center, corresponding to an equatorial diameter of 5 mm. In this second phase of the study, measurements were made at equatorial diameters of 6 mm and 7 mm in both adult and aged lenses. Two structural parameters were measured: average length of straight fibers and cross sectional area. Complete results are presented in Tables 3 and 4.

**Table 3:** Average Length of Straight Fibers ± SEM

	Equatorial Diameter = 7 mm			Equatorial Diameter = 6 mm		
Age Group	Avg. Length (mm)	Range	n	Avg. Length (mm)	Range	n

Adult	6.72 ± 0.17	6.15–7.41	7	6.01 ± 0.20	5.35–6.59	6
Aged	6.94 ± 0.10	6.48–7.23	7	5.97 ± 0.07	5.60–6.19	7
p Value	0.830	---------	---	0.302	---------	---

**Table 4:** Average Cross-Sectional Fiber Area

	Equatorial Diameter = 7 mm			Equatorial Diameter = 6 mm		
Age Group	Avg. Area (μm^2^)	Range	n	Avg. Area (μm^2^)	Range	n

Adult	17.31 ± 2.37	8.60–28.23	7	17.04 ± 2.37	10.78–27.10	6
Aged	8.98 ± 0.63	5.61–10.55	7	10.29 ± 0.43	7.99–11.41	7
p Value	0.005*	---------	---	0.011*	---------	---

* Statistically significant

**Figure 6:**
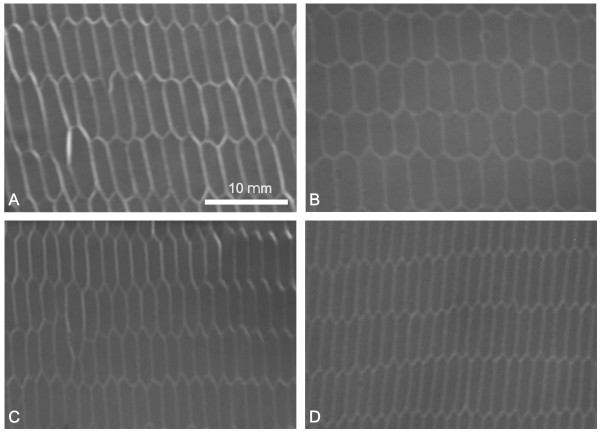
Representative light micrographs from thick (1–2 μm) sections of cross-sectioned cortical fiber cells. A. Adult lens at 7 mm equatorial diameter. B. Adult lens at 6 mm equatorial diameter. C. Aged lens at 7 mm equatorial diameter. D. Aged lens at 6 mm equatorial diameter. Cross sectioned fibers from aged rabbit lenses were noticeably and significantly smaller than those from adult rabbit lenses.

The average length of straight fibers was not significantly different between the adult and aged lenses at either 6 mm or 7 mm equatorial diameter. However, measures of average cross-sectional fiber area showed significant difference at equatorial diameters of both 6 mm and 7 mm (Table 4). At 7 mm equatorial diameter, adult lenses had an average cross sectional area of 17.31 μm^2 ^as compared to 8.98 μm^2 ^in aged lenses (p = 0.005). Similarly, at 6 mm equatorial diameter, adult lenses had an average cross sectional area of 17.04 μm^2 ^as compared to 10.29 μm^2^in aged lenses (p = 0.011). Representative light microscopic images of cortical fiber cells are presented in Figure 6 and demonstrate the perceptible decrease in cross-sectional area as a function of age (compare panel A to panel C and panel B to panel D).

Average fiber volume was estimated by multiplying average fiber length by the average cross-sectional area for each specimen. As expected, estimates of fiber volume were similar to the findings for average cross-sectional area. Complete fiber volume estimates are presented in Table 5. At an equatorial diameter of 7 mm, average fiber volume was significantly less (p = 0.010) in aged lenses when compared to adult lenses. Similarly, at 6 mm equatorial diameter, average fiber volume of aged lenses was significantly less (p = 0.016) in aged vs. adult lenses.

**Table 5:** Average Estimated Fiber Volume

	Equatorial Diameter = 7 mm		Equatorial Diameter = 6 mm	
Age Group	Avg. Volume (μm^3^)	Range	Avg. Volume (μm^3^)	Range

Adult	117377.00 ± 17581.28	58652–196745	103569.44 ± 15882.05	57705–167222
Aged	62064.68 ± 4044.05	40504–71566	61399.36 ± 2334.65	49379–69539
p value	0.010*	---------	0.016*	---------

* Statistically significant

## Discussion

This investigation quantified the amount of scatter and fiber compaction in rabbit lenses as a function of age. Our data confirmed the results of a prior study[19] which showed a significant increase in overall scatter as a function of age. In the present study the scatter data was analyzed regionally, revealing that while all regions of aged rabbit lenses had significantly greater scatter than the corresponding regions in adult lenses, the age-related difference was somewhat greater in the region corresponding to the inner cortical fibers. Furthermore, the morphometric results documented that significant age-related fiber compaction occurred in the inner cortex. Taken together, these data establish a tentative correlation between compaction and scatter in pre-cataractous aged rabbit lenses.

Because all regions of aged rabbit lenses had significantly increased scatter, it is likely that factors other than compaction contributed to the observed increase in scatter. These include changes at both the cellular and molecular levels which correspond, respectively, to large and small particle scatter [42]. Specifically, at the molecular level, posttranslational protein modifications such as glycation, phosphorylation, deamidation, and oxidation [1-3] can alter protein conformation, which may ultimately result in cross-linking and aggregation[4] in the cytoplasm. At the cellular level, one or more structural alterations such as fiber folds and breaks [5], multi lamellar bodies[6,7], extracellular space deposits[8,9], malformed or excessive suture sub-branches[10] and syneresis and micro-phase separation of fiber cytoplasm [11-14] may be present in aged lens fibers, and would be expected to play a role in the increased age-related scatter.

As expected, rabbit lenses in this study demonstrated an age-related change in both equatorial and axial dimensions. Specifically, average equatorial diameter was 6.08% larger and average A-P axis length was 5.74% larger in aged as compared to adult lenses. This increase is larger than anticipated with respect to our prior results [19], however it is consistent with earlier published data showing that between 2–4 years of age rabbit lenses have not yet reached a growth plateau [43]. It is likely that the increase in overall lens thickness (A-P axis) is another component of the observed increase in overall scatter in the lenses in this study, since medium thickness has an impact on transmission.

Observational studies of whole lenses from a variety of etiologies have provided evidence that compaction is an ongoing process affecting fibers at all stages of lens development, growth and aging[18,19,44-46]. Additionally, ultrastructural investigations have shown that compaction has specific, measurable effects on several aspects of lens fiber structure. In particular, compaction along the polar axis of the lens alters the membrane architecture of fibers, affecting both the overall membrane topology[16,17] and the distribution of intramembrane particles and junctions[47]. This component of compaction has an influence on both the packing density and arrangement (specifically the angle of curvature) of nuclear fibers, and also produces accordion-like folds that reduce overall fiber length. In the present investigation, similar changes were noted in the nucleus of aged rabbit lenses, although they were less pronounced than in aged human lenses. It is likely that the reduced amount of compaction in rabbit lens nuclei as compared to humans is due to several factors, including the several decade age difference between aged humans (60–80 years) and aged rabbits (3.5–4.5 years), the differences in overall lens size, sphericity and fiber arrangement (i.e. line sutures vs. complex star sutures) and biomechanical properties [48-50] that affect compliance, permeability and hardness.

The limited amount of compaction documented in the fetal nuclear region (Table 2) in this study was primarily manifested as a significant decrease in the elliptical angles described by both anterior and posterior segments of inner fetal fibers (located at 1 mm from the lens center). The more acute angles in aged lenses as compared to adult lenses signify that compaction occurred along the polar axis in the inner nucleus. The outer fetal fibers (located at 2.5 mm from the lens center) were not significantly decreased as a function of age. However, careful examination of the data showed that outer fetal fibers from both adult and aged lenses had comparable angles to inner fetal fibers of aged lenses, indicating that they were compacted in both adult and aged lenses. This data suggests that, in rabbit lenses, compaction in the outer FN fibers precedes compaction in the older fibers of the inner FN and EN.

It is well known that the nucleus is harder than the more superficial fiber layers in adult mammalian lenses and that this increase in hardness becomes more pronounced with age [51] as does an increase in nuclear stiffness [52]. Both factors contribute to the loss of nuclear flexibility [49]. Hence, if the lenses in this study were subjected to a limited amount of osmotic stress, it is apparent that the central fibers would be less susceptible to osmotic pressure than the peripheral fibers. With aging, the response to osmotic factors would be further lessened. From this it can be seen that adult rabbit lens nuclei would be slightly more susceptible to osmotic stress (such as aldehyde shrinkage and membrane deformation) than aged rabbit lens nuclei. Therefore, our data, which showed only moderate evidence of compaction in the lens nuclei as a function of age (decreased elliptical angles and increased 'accordion folds'), may actually underestimate the age-related differences.

Definitive evidence of fiber compaction in the inner cortex was documented in the present investigation. Specifically, the average cross-sectional area and the estimated volume of cortical fibers were significantly reduced as a function of age (Tables 4 and 5). However, the average cortical fiber length was virtually identical in adult and aged lenses (Table 3). The loss of cell volume without a change in overall length suggests that cortical fiber compaction occurred as a result of compression along the equatorial plane rather than along the polar axis as seen in nuclear regions. The 'thinning' or reduction of rabbit lens fiber thickness (evident in Figure 6) is consistent with images from aged human lenses showing extremely flattened, compressed fibers in the most superficial nuclear regions[9,15,37]. However, it should be noted that the thinning of human lens fibers is much more extreme than in rabbit lenses, resulting in an inability to distinguish individual adult nuclear fiber profiles at the LM level. It is possible that the fibers located near the cortical-nuclear interface in both human and rabbit lenses are more susceptible to inward compressive forces along the equatorial plane because they are less rigid[22,53] than the older, more centrally located nuclear fibers. In fact, the entire lens nucleus hardens with both aging and cataract formation[46,52], resulting in decreased compliance[54]. These age-related alterations may have been factors affecting the observed structural changes in the lens nuclei. Specifically, because lenses were removed from the support of the ciliary muscle and suspensory ligaments before fixation, a small degree of overall rounding probably occurred. However, because aged lens nuclei are generally more rigid, they display decreased flexibility[48]and so would be expected to exhibit less rounding upon detachment from the zonules and ciliary body than the younger adult lenses. This would tend to lead to underestimation of the differences between age groups.

One possible cause for the significant reduction in fiber volume in the present study is water loss. In fact, Raman spectroscopy of both rabbit and human lenses has shown that the water content decreases dramatically from the superficial cortex to the deep cortex[55]. Unfortunately, there is no clear agreement about water content in the nucleus of aged and cataractous lenses, with some analyses showing decreased water in the nucleus [50,56-59], some finding no change with aging or cataract state[46,52] and yet others showing an increase in nuclear water content with aging[60] and cataract [61].

It is likely that changes at the molecular level, such as extensive protein cross-linking and/or formation of high-molecular weight protein aggregates, may be implicated in changes to lens physical properties such as hardening and decreased compliance[1,62] as well as in fiber compaction, since protein aggregation alters the interactions between cytoplasmic proteins and membranes [63].

In this investigation, laser scan analysis revealed that significantly more scatter was present in all regions scanned (up to 10 mm equatorial diameter) as a function of age. As stated above, molecular rearrangements leading to protein cross linking and aggregation, which are known to occur throughout all developmental regions of aged lenses [64-71], probably contributed to the overall change in transmittance through rabbit lenses. In the present study, preparative constraints precluded structural analysis of the superficial cortex which could have elucidated the relationship between cortical scatter and compaction. It is clear that such an analysis must be a priority in future investigations to determine if lens fiber compaction also contributes to the observed age-related increase in scatter within the cortex.

## Conclusion

It is well established that the precise arrangement and packing of fibers in a crystalline array contributes to lens transparency [10,20] and conversely, that any disruption or modification of this exact fiber arrangement adversely affects lens function[72,73]. Because age-related compaction alters fiber ultrastructure (impacting both the packing density of fibers and their membrane topology) it is logical to assume that those compaction-induced changes contributed to the degradation of the optical properties of the lenses in this study.

Our results do not minimize the importance of oxidation (which affects both cytoplasmic and membrane proteins), the development of cross-linked, high molecular weight protein aggregates, or the various ultrastructural fiber alterations that have been documented in both aged normal lenses and in age-related nuclear cataracts. Rather, the confirmation that aged animal lenses show similar, albeit less dramatic, changes to aged human lenses, particularly in the inner cortex and outer nuclear regions, underscores the fact that compaction is one of an array of changes that precedes and may, in fact, contribute to the development of age-related cataract.

## Competing interests

The author(s) declare that they have no competing interests.

## Authors' contributions

SA carried out portions of the structural assessment and statistical analysis of morphometry and also wrote the draft manuscript. SD participated in the laser scan analysis and performed parts of the structural assessment. WA participated in the laser scan analysis and aspects of the design. KA conceived and designed the study, participated in the laser scan analysis, structural assessment, and statistical analysis and also assisted in manuscript preparation. All authors read and approved the final manuscript.

## Pre-publication history

The pre-publication history for this paper can be accessed here:


